# Inequalities in health care among patients with type 2 diabetes by individual socio-economic status (SES) and regional deprivation: a systematic literature review

**DOI:** 10.1186/1475-9276-13-43

**Published:** 2014-06-02

**Authors:** Olga Grintsova, Werner Maier, Andreas Mielck

**Affiliations:** 1 Helmholtz Zentrum Muenchen, Institute of Health Economics and Health Care Management, PO Box 1129, Neuherberg D-85758, Germany

**Keywords:** Type 2 diabetes mellitus, Health care, Socio-economic status, Regional deprivation, Review

## Abstract

**Introduction:**

Quality of care could be influenced by individual socio-economic status (SES) and by residential area deprivation. The objective is to synthesize the current evidence regarding inequalities in health care for patients with Type 2 diabetes mellitus (Type 2 DM).

**Methods:**

The systematic review focuses on inequalities concerning process (e.g. measurement of HbA1c, i.e. glycolised haemoglobin) and intermediate outcome indicators (e.g. HbA1c level) of Type 2 diabetes care. In total, of n = 886 publications screened, n = 21 met the inclusion criteria.

**Results:**

A wide variety of definitions for ‘good quality diabetes care’, regional deprivation and individual SES was observed. Despite differences in research approaches, there is a trend towards worse health care for patients with low SES, concerning both process of care and intermediate outcome indicators. Patients living in deprived areas less often achieve glycaemic control targets, tend to have higher blood pressure (BP) and worse lipid profile control.

**Conclusion:**

The available evidence clearly points to the fact that socio-economic inequalities in diabetes care do exist. Low individual SES and residential area deprivation are often associated with worse process indicators and worse intermediate outcomes, resulting in higher risks of microvascular and macrovascular complications. These inequalities exist across different health care systems. Recommendations for further research are provided.

## Introduction

Type 2 diabetes mellitus (Type 2 DM) is one of the leading causes of death in the world with constantly increasing prevalence: in 2008, the prevalence was estimated to be 10% among adults aged 25+ years [1]. The rising burden of diabetes is associated with a constant increase in its complications, causing rising disability and booming health care costs ranging from 2.5% to 15% of annual health care budgets [2]. Good diabetes care is crucial to delay these complications. Good glycaemic control results in a reduction of complications and better patient outcomes. The data from the United Kingdom Prospective Diabetes Study (UKPDS) concerning patients with Type 2 DM suggested, for example, a decreased risk of 25% in retinopathy and nephropathy for every reduction of 1% in HbA1c [3].

Different patient groups are affected unequally by Type 2 DM. There are large differences, for example, concerning gender, age and race. Socio-economic status (SES) may influence access to and quality of care, social support and availability of community resources. It may also influence diabetes-related knowledge, communication with providers, treatment choices and the ability to adhere to recommended medication, exercise and dietary regimens [4,5]. Thus, low SES could be associated with multiple risks. First, epidemiological studies have repeatedly confirmed the inverse association between Type 2 DM and SES [6-9]. Also, regional deprivation (often used as a proxy for individual SES) was shown to have an independent influence on the incidence and prevalence of diabetes mellitus [10]. Second, some analyses focusing on patients with Type 2 DM indicate that health care could be worse for low SES groups, but this has rarely been studied in a systematic way.

To date, there is just one systematic review regarding inequalities in health care among patients with Type 2 diabetes by individual socio-economic status (SES) and regional deprivation. Ricci-Cabello et al. (2010) [11] focused on countries from the Organisation for Economic Cooperation and Development (OECD). They looked at differences by gender, ethnic group and SES, and for SES they reported results concerning diagnosis, control of diabetes and access to health services (four, seven and five studies respectively). They conclude that the results point towards health inequalities favouring higher SES groups, and that there is a great need for more empirical research. Their review covers publications up to 2007, and a number of papers have been published since. Thus, it was our objective to synthesize the current evidence, focusing on process and intermediate outcome indicators of health care for patients with Type 2 DM and on differences by individual SES and residential area deprivation.

## Methods

In November and December 2012, we conducted a systematic literature search in PubMed for original studies published in English, German, French or Spanish. A combination of the following key words was used: diabetes, diabetes Type 2, quality of care, management, care, control, soci*, socio*, inequ*, differ*, dispar*, regio*, depriv* (and their translations) in title and abstract. The American Diabetes Association (ADA) guidelines for good diabetes control and quality of care were changed in 2002 (with subsequent improvements in 2005 and 2011) [12,13] and, in 2005, the first Global Guideline for Type 2 Diabetes was published by the International Diabetes Federation (IDF) [14], complicating the comparison between previous and later studies. In order to improve the compatibility between studies, the time frame for the search was restricted to papers published between 1 January 2002 and 13 December 2012.

**Figure 1:**
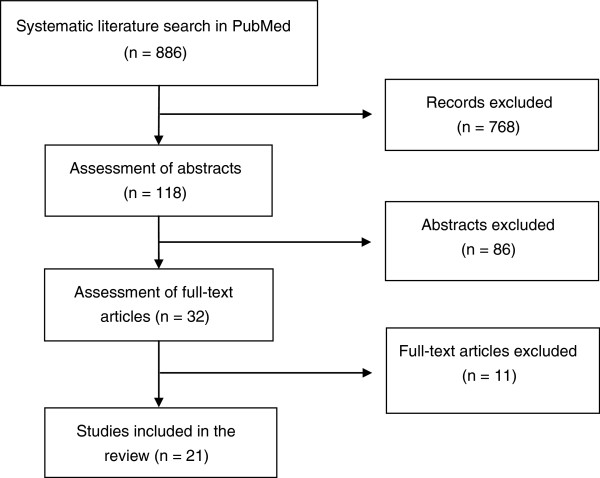
Selection of articles.

Altogether, 886 publications were eligible for further screening (see Figure 1). Titles and abstracts were screened, using the inclusion criteria outlined below and the PRISMA statement for reporting the results [15]. The references from selected articles were screened for further potentially relevant studies.

An article was included in the review: (a) if the study population comprises Type 2 DM patients or a mixed group of Type 1 and Type 2 DM patients; (b) if quantitative analyses are presented concerning process and intermediate outcome indicators of health care; (c) if individual SES and/or residential area deprivation is included as an independent variable. We focused the review on process indicators such as assessment of HbA1c (glycolised haemoglobin), BP (blood pressure), BMI (body mass index), and on intermediate outcome indicators such as quantitative measurement of HbA1c, BP and/or lipids in the blood. All these indicators relate directly to health care provision, they are usually well documented and show relatively short-term results. They are influenced by other factors as well, of course, including individual health behavior and social support. Health care plays an important role, though, and most guidelines for good diabetes control include targets such as reduction of high BP, smoking cessation and reduction of high body weight.

Quality of care could also be assessed by outcome indicators less closely associated with health care, such as complications, hospital admissions or cardiovascular disease (CVD) mortality [16]. Results concerning these ‘other outcome indicators’ are mentioned below just briefly. The exclusion criteria are: studies restricted to Type 1 DM and studies just focusing on social disparities by race, ethnicity and nationality or migration status. These social disparities could be closely linked to individual SES and residential area deprivation, of course, but we still believe that they touch on a different topic.

Different information was extracted from the studies finally selected, based on the data extraction sheet proposed by Aveyard [17]; the results are presented in Tables 1 and 2. We also tried to assess and compare the methodological quality of the studies; this task proved to be quite challenging. The studies included in the review were conducted in very different settings (e.g. hospital, primary care), using a wide variety of methodological approaches (e.g. national or local registry data, practice-level data, patient survey). It is hardly possible to compare them in a common scheme that would account for all these differences. Finally, we used an adopted version of the EPHPP (Effective Public Health Practice Project) quality assessment tool [18]. It includes different components (evaluation of selection bias, study design, confounders, blinding, withdrawals and drop-outs, intervention integrity). Each component must be rated as providing ‘strong’, ‘moderate’ or ‘weak’ evidence; the final scale summarizes these ratings. We applied relatively strict criteria: to be rated as ‘strong’ evidence, a study must have no weak component and at least three out of six strong ones. The criteria for ‘moderate’ evidence are: one ‘weak’ component, or no ‘weak’ and less than three ‘strong’ ratings. All other studies were rated as ‘weak’. Screening, information extraction and assessment was first performed by OG and WM, in case of disagreement a joint decision was reached together with AM.

**Table 1:** **Quality of health care for people with type 2 diabetes: process and intermediate outcome indicators**^**a**^

	**Process indicators: delivery of assessment**							**Intermediate outcome indicators: quantitative measurement**^**b**^		
**Ref.**	**HbA1c**	**Lipids**	**Retino- pathy**	**BP**	**BMI**	**Smoking**	**Micro-albuminuria**	**HbA1c**	**Lipids**	**BP (mmHg)**
[19]	X	X	X	─	─	─	─	─	─	─
[20]	X	─	─	X	X	─	─	Mean	Mean^1^	Mean
[21]	─	─	─	─	─	─	─	Mean	─	Mean
[22]	─	─	─	─	─	─	─	Mean	─	─
[23]	─	─	─	─	─	─	─	Mean	> 2 g/L^3^	─
[24]	─	─	─	─	─	─	─	≤ 7.4%	≤ 5 mmol/L^1^	≤ 145/85
[25]	X	X	X	─	─	─	─	Mean	Mean^2^	> 140/90
[26]	─	─	─	─	─	─	─	Mean	< 100 mg%^2^	─
[27]	X	X	X	─	─	─	X	─	─	─
[28]	X	X	X	X	X	X	X	≤ 7.4%; ≤ 10%	≤ 5 mmol/L^1^	≤ 145/85
[29]	X	X	─	X	─	X	─	≤ 7.4%	≤ 5 mmol/L^1^	≤ 140/80
[30]	─	─	X	─	X	X	X	< 7.5%; < 10%	≤ 5 mmol/L^1^	< 145/85
[31]	X	X	─	─	─	─	─	─	─	─
[32]	─	─	X	─	─	─	─	Mean	≥ 5^4^	≥ 140/90
[33]	X	X	─	─	X	X	─	Mean; ≤ 7.5%	─	─
[34]	─	─	─	─	─	X	─	≥ 6.5%	≥ 2.58 mmol/L^2^	─
[35]	X	X	X	X	X	X	X	≤ 7.4%; ≤ 10%	≤ 5 mmol/L^1^	≤ 145/85
[36]	─	─	─	─	─	─	─	≤ 6.5%	≤ 5.2 mmol/L^1^	≤ 140/90
									> 1.1–1.4 mmol/L^2^	
[37]	X	─	X	X	─	─	X	< 7.0%	< 100 mg/dL^2^	< 130/80
[38]	X	X	─	X	X	─	─	≥ 9%	< 100 mg/dL^2^	─
[39]	X	X	X	X	X	X	─	≤ 7.0%	≤ 2.6 mmol/L^2^	≤ 130/80

^1^Total cholesterol; ^2^LDL; ^3^TG; ^4^Total cholesterol/HDL-coefficient.

^a^other process and outcome indicators are just mentioned in the text (see ‘Results’).

^b^mean values or cut-off values for ‘good quality health care’.

HbA1c, glycolised haemoglobin; BP, blood pressure; BMI, body mass index.

**Table 2:** Characteristics of the studies

**Reference**	**- Country**	**Assessment of individual level socio-economic status (SES)**	**Assessment of regional deprivation**	**Main results**	**Main conclusion**
	**- n**				
	**-% women**				
	**- type of diabetes**				
	**- age (years)**				
	**1**	**2**	**3**	**4**	**5**
Arday D.R. et al. [19]	- USA	–	ZQ score (household income; education; occupation; home value)	- HbA1c tests (sign.), eye examinations (sign.) and lipid profile measurements (sign.) less likely for patients living in the most deprived regions	- Worse diabetes care in the most deprived states
	- n = 1,941,517				
	- 54.4%				
	- T1D + T2D				- Substantial reduction in variation between states concerning diabetes care after adjustment for residents’ characteristics (ZQ score)
	- 18–75				
	- 67.5 (median)				
Bachmann M.O. et al. [20]	- UK	Educational level, individual income	–	- Higher HbA1c values (sign.), more non-compliance (sign.), retinopathy (trend) and less hospital attendance (sign.) among patients with low education	- Larger burden of morbidity for patients with low SES, but less hospital care
	- n = 555				
	- 47%				
	- T1D + T2D				
	- 65 (mean)				
				- More complications among patients with low education and low income (sign.)	
				- More negative effects on social life (sign.) and personal life (sign.) among patients with low education and low income	
Bäz L. et al. [21]	- Germany	Index based on educational level, occupational status and income	–	- Increasing HbA1c (sign.) and BMI (sign.) with decreasing SES	- Worse diabetes control for patients with low SES
	- n = 555			- No sign. association between BP and SES	
	- 50.2% (T1D)				
	45.5% (T2D)				
	- T1D + T2D				
	- 56.1 (mean)				
Bebb C. et al. [22]	- UK	–	Townsend score	- Higher HbA1c values for patients living in the most deprived regions (sign.) and for practices with many patients living in the most deprived regions (sign.)	- Worse glycaemic control in the most deprived regions
	- n = 1,534				
	- 41%				
	- T2D				- Effect of regional deprivation most important for extreme deprivation
	- 18–80				
	66.2 (median)				
				- Variation between practices concerning HbA1c values largely explained by percentage of patients living in the most deprived regions	
	1	2	3	4	5
Bihan H. et al. [23]	- France	EPICES score	–	- Higher HbA1c values (sign.), more complications such as retinopathy (sign.) and neuropathy (sign.) and fewer 1-day hospitalizations (sign.) in patients with low SES	- Worse glycaemic control and more microvascular complications in patients with low SES
	- n = 135				
	- 48.8%				
	- T1D + T2D				
	- 59 (mean)				
Bottle A. et al. [24]	- UK	–	IMD	- Age group 25–59 years: higher total hospital (sign.) and ketoacidosis admission rates (sign.) for patients living in the most deprived regions	- Worse diabetes control, more hospital admissions and lower quality of health care in the most deprived regions
	- n = 1,760,898				
	- (unknown)				
	- T1D + T2D;				
	- 25–59			- Age group ≥60 years: higher total hospital admission rates (sign.), lower total quality score (not sign.) and lower HbA1c control score (not sign.) for patients living in the most deprived regions	
	25–59 (main group, 47.9%)				
Brown A.F. et al. [25]	- USA	Educational level, income	–	- Small SES differences concerning six out of seven process indicators, i.e. lipid profile measurement, foot examination, aspirin advice or use, influenza vaccination (all not sign.) and in two out of three intermediate outcomes, i.e. HbA1c control (not sign.) and LDL control (sign.)	- Worse diabetes control for patients with low SES concerning glycaemic control and dilated eye examinations
	- n = 7,456				
	- 53%				
	- T1D + T2D				
	- 60.4 (mean)				
				- Higher HbA1c values (sign.) and fewer dilated eye examinations (sign.) in low SES group	
Geraghty E.M. et al. [26]	- USA	–	Differences by income, educational level, unemployment (GIS analyses)	- Higher HbA1c values for patients living in more deprived regions (not sign.)	- Tendency for worse glycaemic control in most deprived regions
	- n = 7.288				
	- 47.4%				
	- T1D + T2D			- No association between regional deprivation and LDL control	
	- 62 (mean)				
Gnavi R. et al. [27]	- Italy	Educational level, income	–	- Fewer measurements of HbA1c (not sign.) and eye examinations (not sign.) among patients with low education	- Most SES differences not statistically significant
	- n = 33,453				- SES differences in health care processes favouring the disadvantaged group
	- 49.4%			- Fewer ECG measurements (not sign.) and visits to diabetologists (not sign.) among patients with low education or low income	
	- T1D + T2D				
	- 65–74				
	65–75 (main group 34.3%)				
	1	2	3	4	5
Gray J. et al. [28]	- UK	–	IMD	- Small difference concerning health care process between most and least deprived regions	- Tendency for worse health care in the most deprived regions
	- n = 6,035				
				- Higher values of HbA1c (not sign.) and BP (not sign.) for patients living in the most deprived regions	
	- 48.3%				
	- T1D + T2D				
	- 65–74 (main group, 26.6%)				
Guthrie B. et al. [29]	- UK	–	Carstairs deprivation score	- Less complete process indicators of good quality health care for patients living in the most deprived regions (sign.)	- No consistent association between quality of care and regional deprivation
	- n = 10,191				
	- 47.1%				
	- T2D				
	- 67.8 (median)				
				- Higher percentage of smokers for patients living in the most deprived regions (sign.)	
Hippsley-Cox J. et al. [30]	- UK	–	Townsend score	- Higher BMI (sign.), more smokers (sign.), less neuropathy (sign.), microalbuminuria (sign.) and eye examinations (sign.), fewer flu vaccinations for patients living in the most deprived regions	- Worse process of care and worse outcomes in the most deprived regions
	- n = 53,678				
	- (unknown)				
	- T1D + T2D				
	- >16				
				- Higher HbA1c values (sign.) and BP values (sign.)	
Hsu C.C. et al. [31]	- Taiwan	Income	–	- More severe diabetes symptoms (sign.), fewer visits to diabetes clinic (sign.) and to ambulatory diabetes clinic (sign.) in low SES group	- Higher probability of hospital-diagnosed diabetes following severe symptoms and smaller likelihood of receiving recommended diabetes check-ups for patients with low SES
	- n = 1,462				
	- 39.3–50.4%				
	- T2D				
	- > 20				
				- Fewer tests for HbA1c (sign.), LDL (sign.), triglycerides (sign.) and fewer examinations for retinopathy (sign.) in low SES group	
Icks A. et al. [32]	- Germany	Helmert index (based on educational level, occupational status, income)	–	- Unfavourable process (retinopathy screening, foot examination, diabetes education, lipid and BP control, all not sign.) and outcome (HbA1c, not sign.) indicators in low SES group	- Preliminary indications of less than good health care especially for patients with low SES
	- n = 149				
	- 46%			- Less knowledge about the term ‘HbA1c’ in low SES group (sign.)	
	- T2D				
	- 62 (mean)				
	1	2	3	4	5
James G.D. et al. [33]	- UK	–	Townsend score	- Higher values of HbA1c in most deprived regions (sign.)	- Worse glycaemic control in deprived regions, independent of other social factors such as ethnicity
	- n = 24,111				
	- 47%				
	- T2D				
	- 52.6 (median)				
Larranaga et al. [34]	- Spain	–	Index from Basque Institute of Statistics	- Higher values of LDL (sign.) and HbA1c (sign.) in most deprived regions	- Worse health care and higher prevalence of clinical complications in most deprived regions
	- n = 2,985				
	- 52%				
	- T2D			- Higher risk of macroangiopathy complications (sign.) and higher risk of retino-, neuro- and nephropathy (not sign.) in most deprived regions	
	- ≥ 24				
				- More consultations per year in most deprived regions (sign.)	
Millet C. et al. [35]	- UK	–	IMD	- Lower rates of reaching health care targets concerning HbA1c, BP and cholesterol in most deprived regions	- Worse intermediate outcomes in most deprived regions
	- n = 1,852,762				
	- unknown				
	- T1D + T2D				
	- > 18				
Reisig V. et al. [36]	- Germany	Helmert index (based on educational level, occupational status, income)	–	- Among patients with T2DM and myocardial infarction: lower probability of reaching HbA1c target (sign.) and higher prevalence of retinopathy (sign.) in low SES group	- Worse glycaemic control and more complications for patients with low SES
	- n = 373				
	- 31.9%				
	- T2D				
	- 68.1 (median)				
Shani M. et al. [37]	- Israel	Income	–	- Lower probability of reaching HbA1c target (sign.) and LDL target (sign.) in low SES group	- Worse glycaemic control for patients with low SES
	- n = 18,316				
	- 50.3%				
	- T1D + T2D				
	- 65.5 (median)				
	1	2	3	4	5
Wilf-Miron R. et al. [38]	- Israel	–	Ranking based on educational level, employment, income, housing density	- ‘Optimal follow-up’ (sign.) is more likely in most deprived regions	- Process indicators: better health care quality in the most deprived regions
	- n = 74,953				
	- 46.1%				
	- T1D + T2D			- Appropriate LDL control (not sign.) and achieving HbA1c target (sign.) less likely in most deprived regions	- Intermediate outcome indicators: worse health care quality in the most deprived regions
	- 59 (mean)				
Wong K.W. et al. [39]	- China	Fee for service waiver (yes/no)	–	- Recording of cholesterol (sign.) and of BMI (sign.), screening for nephropathy (sign.) and retinopathy (sign.) less likely in low SES group	- Process indicators: worse health care for patients with low SES
	- n = 1,970			- Achievement of BP target (sign.) and HbA1c target (not sign.) less likely in low SES group	- Intermediate outcome indicators: worse health care for patients with low SES
	- 55.1%				
	- T2D				
	- 63.2 (median)				

Sign.: 5% level.

T1D, Type 1 diabetes.

T2D, Type 2 diabetes.

## Results

Based on the inclusion and exclusion criteria listed above, 32 articles were identified for full-text review (see Figure 1).

Eleven of them were excluded for different reasons (no data on individual SES or regional deprivation, no data on quality of health care, or no analysis of the association between these two topics).Most of the remaining 21 papers are from Western European countries, i.e. from the UK (n = 8), Germany (n = 3) and one each from France, Italy and Spain. There are also three papers from the USA, two from Israel and one each from Taiwan and China. They comprise cross-sectional (n = 12) and cohort studies (n = 9), and most used the retrospective data (n = 17). Some (n = 8) are limited to Type 2 DM patients; the others use a mixed sample of both Type 1 and Type 2 DM patients. Sample size varies between 135 and 1,941,517 patients; mean (or median) age varies between 52.6 and 68.1 years. The process and intermediate outcome indicators (including cut-off points of good control) are presented in Table 1. The main characteristics and results of the studies are summarized in Table 2.

### Individual SES and regional deprivation

Individual SES was assessed in 10 studies, mostly by income [31,37,39] and educational level. These two indicators were used side by side [20,25,27] or combined with the indicator ‘occupational status’ in order to build an index of SES [21,23,32,36]. The association between regional deprivation and quality of health care was evaluated in 11 studies. In five of them (all from the UK), this was the primary objective of the study, using different tools to assess regional deprivation, i.e. the Townsend score [22,30,33], the Carstairs deprivation score [29] and the Index of Multiple Deprivation (IMD) [24,28,35]. In the other three studies, regional deprivation was used as a proxy for individual SES. In one study from the USA, the ZQ score (zip quality ranking of participant’s residence, based on economic indicators for the state) was used as a very broad measure for individual SES [19]. In the other three studies from the USA, Spain and Israel, individual SES was assessed by neighbourhood SES using census data combined with GIS (geographic information system) [26] or local standard of living data [34], or by using the economic ranking of the geographical sub-district where the patients lived [38].

### Quality of care indicators

Three groups of process indicators can be distinguished: (a) delivery of clinical parameter assessments (e.g. concerning HbA1c, lipid profile, BP, BMI, retinopathy examinations by the physician); (b) smoking, as it is well established that Type 2 DM patients should not smoke; (c) visits to a GP (General practitioner) or a diabetologist. The intermediate outcome indicators are: numerical value of clinical parameters (e.g. concerning HbA1c, lipid profile, BP, BMI) as compared with established cut-off points for good control.

The process indicators used most often are presented in Table 1. Further process indicators included, for example, complications [20,25,30-32,35], creatinine [23,28-31,35], flu vaccination [25,28,30,35], microalbuminuria test [27,28,30,34,36], pulses [28,30,35], attendance at general practices [20], angiotensin converting enzyme inhibitors received in the presence of proteinuria or microalbuminuria [30,35], adherence [20], use of or advice to use aspirin [25], ECG [27], proportion of diabetic patients who were diagnosed through hospitalization [31], diabetes education and knowledge of ‘HbA1c’ definition [32], sedentary lifestyle [34]. Several composite process indicators have been used as well: based on a patient questionnaire [20], on an assessment of HbA1c and at least two assessments out of three (eye, total serum cholesterol, microalbuminuria) per annum [27], on the performance of all tests out of seven (HbA1c, LDL [low-density lipoprotein]), nephropathy monitoring, eye and foot examinations, BP, BMI recorded during the past 12 months) [39].

Concerning the intermediate outcome indicators, the cut-off points for good control vary greatly. For HbA1c, for example, they vary from ≥ 6.5% [34] or ≤ 6.5% [36] to ≥ 9% [38] or ≤ 10% [28]. Based on international guidelines [40], the cut-off points for lipids used most often are LDL ≤ 100 mg% or ≤ 2.6 mmol/L [25,26,34,37-39]. For total cholesterol, many studies use ≤ 5 mmol/L as the cut-off point for good control [24,28-30,35]. Other measures are used as well, e.g. concerning hypertriglyceridaemia [23], coefficient ‘total cholesterol/HDL’ [32] and HDL [36]. BP level was used in 11 studies: the cut-off points vary from < 130/80 [37] to ≤ 145/85 [24].

Looking also at other outcome indicators, some studies assessed health care quality by the presence of complications [23,32,36], high creatinine level [23,36], presence of anaemia [23], total and ketoacidosis hospital admissions [24,27], absence of obesity (BMI ≥ 30 kg/m^2^) [36], proportion of non-smokers [29,36] and proportion of patients with high physical activity (more than 1 hour per week) [36]. In all but two studies [21,35], the results were adjusted for age and sex. Also, many results were adjusted for diabetes duration [20-23,25,29,31,32,34,36,39] and BMI [21,23,33,37,39].

### Associations with individual SES

Most studies show that lower individual SES is associated with worse diabetes care. These disparities are reported for both process and outcomes of diabetes care. Concerning process indicators, most of these differences are statistically significant [31,39] or show a similar trend [20,25,32]. Low SES patients visit diabetes clinics and ambulatory care facilities less often [31], but they are also more likely to visit a diabetologist [27], probably reflecting a greater need for better diabetes control. Two studies show no major difference by individual SES [37] or only slight differences in dilated eye examination rates [25]. In one study, the low SES group was favoured concerning health care processes [27].

Turning to the intermediate outcome indicators, the results differ substantially by the indicator used. In all studies looking at individual SES and glycaemic control, lower SES patients have higher HbA1c values than other patients, either significantly [20,21,23,25,36,37] or as a similar trend [32,39]. Socio-economic position is important even at the practice level: a higher proportion of patients with low SES registered at a practice is significantly associated with a lower percentage achieving good control (HbA1c < 7 mg%) [37]. The association between individual SES and BP is investigated in seven studies, and no significant association is found in six of them [20,21,25,32,36,37]. In the other study, the chance of low SES patients achieving the BP target is reduced by about 30% (p <0.05) [39]. The achievement of clinical targets for lipid control is assessed in seven studies, and no significant association with individual SES is found in five of them [20,23,32,36,39]. In the other two studies, low SES is significantly associated with worse lipid control [25,37]. Concerning the other outcome indicators, it has also been shown that low SES patients are more likely to have complications such as retinopathy [20,23,36] and nephropathy [20].

### Associations with regional deprivation

In most of the studies reviewed here, high regional deprivation is associated with worse diabetes care. Concerning process indicators, the results are somewhat contradictory. The most deprived group is likely to have the clinical parameters measured least often; these differences are significant [19,29,30] or point into the same direction [28,35]. There are exceptions though. One study shows that optimal follow-up (i.e. all process indicators recorded) is significantly associated with low regional deprivation [38]. In another study, GP consultations are significantly higher in the most deprived region [34]. It can be hypothesized that ‘consultation efficiency’ is especially low for low SES patients, that they need more visits than high SES patients for achieving the same target.

Concerning intermediate outcome indicators, the association with HbA1c is investigated in 10 studies. In nine of them, higher regional deprivation is associated with higher HbA1c values, and patients living there achieve the glycaemic control targets less often; these differences are statistically significant [22,30,33,34,38] or show a similar trend [24,26,28,35]. No difference between deprivation groups was found in just one study [29]. The achievement of BP targets is investigated in five studies, and in three of them the high deprivation group shows more unfavourable results, either significantly [30] or as a trend [28,35]. No association is found in two studies [24,29]. Lipid control is investigated in eight studies, and in four of them no significant association is found [24,28-30]. In the other studies, there was a clear trend indicating that patients living in deprived areas [26,34,38] or visiting practices in deprived areas [35] are less likely to achieve the lipid profile control targets. Concerning the other outcome indicators, patients from the most deprived regions often experience a greater burden of disease and show significantly higher risks of complications [34] and diabetes-related hospital admissions [24]. Also, the proportion of smokers is higher [29,30] and so is the mean BMI [30].

Following our quality assessment criteria (see ‘Methods’), ‘strong’ evidence is provided by just three studies, they have been conducted in the USA [19], the UK [35] and Israel [38]. The data were taken from registries with high data quality, making problems of selection and reporting bias highly unlikely. The results were adjusted for age, sex, duration of diabetes and other variables, and the process and outcome indicators of health care quality were chosen according to international guidelines [13,14]. The studies show that the values for process indicators are worse in more deprived regions [19], and that intermediate outcome targets (e.g. concerning level of HbA1c) are reached less often in more deprived regions, in the UK [35] as well as in Israel [38].

## Discussion

Studies included here come from a variety of countries, and they vary widely in study design and sample size. About one third use data from registries at the national [19], regional [24,26,27,29-31,35,37,38] or practice level [20,28,33,34]. These registries usually provide high quality data with few missing values, but they mostly lack information on individual SES. Other studies were conducted in a hospital setting [21,23,39], and again data quality should be high. Owing to potential selection bias, it could be difficult, though, to apply the results to the general population. A third group of studies is based on surveys [22,23,32,36]. They might be more representative of the whole population, but they often lack information regarding process indicators. Each study design has its specific advantages and drawbacks, and this also applies to the studies included in this review.

About half of the studies include information on individual level SES, for example by educational level or per capita household income (see Table 2). The other studies include information on regional deprivation, probably often because data on individual SES were not available and regional deprivation was taken as a proxy. Regional deprivation is assessed in different ways, e.g. by the Townsend score [22], the IMD [24] or by a deprivation score developed in Spain [28]. Individual level SES and regional deprivation could both reveal important information, as it could be important to improve health care for specific social groups and/or for specific regions.

The studies reviewed here reflect the problem that there is no unified, internationally accepted definition concerning the quality of health care for people with Type 2 DM. Different approaches to defining and measuring health care quality are used, and it is hardly possible to combine the results in a quantitative meta-analysis. Also, different studies use different sets of indicators. Microalbuminaemia is assessed in just five of these 21 studies, for example, and six studies do not include any process indicator. All but three studies include an intermediate outcome indicator (e.g. comparing HbA1c values with a cut-off value for good glycaemic control), but different cut-off values are used in different studies, making it even more difficult to directly compare the empirical results.

Despite these multifaceted differences between the 21 studies reviewed here, it is still possible to identify some main results: (a) Process indicators of health care quality are often significantly associated with individual SES, i.e. patients with low SES often receive these measurements and assessments less frequently than patients with high SES (e.g. concerning HbA1c, lipids, retinopathy, BP, BMI, smoking status, microalbuminaemia). (b) Individual SES is often significantly associated with poor glycaemic control, i.e. HbA1c values above the recommended clinical target. (c) Concerning BP and lipids above the proposed level, there seems to be no clear association with individual SES. (d) Diabetes-specific complications such as retinopathy and nephropathy seem to be more common among patients with low (as compared with high) individual SES, but these associations have rarely been assessed. (e) The associations with health care quality are mostly less pronounced and consistent if social differences are assessed by regional deprivation instead of individual level SES. (f) Concerning process indicators, some studies indicate that health care quality is worse in more deprived regions, but these associations are statistically significant only in some of them. (g) A similar, but more consistent picture is seen for glycaemic control. HbA1c values indicating poor glycaemic control are found more often in more deprived regions, and in most studies this difference is statistically significant. (h) Some studies indicate that the targets concerning BP and lipids are achieved less often in more deprived regions, but the level of statistical significance is reached only in some of them. (i) Few studies assess the association between regional deprivation and diabetes-specific complications such as retinopathy and nephropathy; they indicate that it is positive and significant.

Some limitations of the review need to be considered. First, we tried to find all relevant studies published up to December 2012, by following a well established, standardized scheme [15], by including all papers irrespective of the statistical significance of their empirical results, by including papers written in different languages (i.e. English, German, French and Spanish) and by performing a counter-checked evaluation of the papers. Our search was confined to publications that could be found in the PubMed database. Following the recommendation from a reviewer we later repeated the search in the EMBASE database (using the identical strategy as outlined above; see ‘Methods’), and we found that all publications identified in EMBASE had also been included in the first group of papers (n = 886) identified in PUBMED (see Figure 1). But of course we cannot rule out the possibility that we have missed relevant publications.

Second, just one third of these studies are clearly restricted to Type 2 DM patients [22,29,31-34,36,39], i.e. the others also include Type 1 DM patients. This should not be a major problem, though, as Type 2 DM accounts for about 90% of all diabetes patients [1]. Of course, there could also be important inequalities concerning health care for type 1 DM [10], but this review focuses as much as possible on type 2 DM. Third, social inequalities can be assessed by a number of indicators besides SES and regional deprivation (e.g. migrant status and ethnicity). These other indicators are not included here, although they might well interact with SES and regional deprivation [5].

The review could be an important contribution to the discussion on health care inequalities among patients with Type 2 DM. Most would agree that these inequalities should be reduced, but to date there seems to be just one review that summarizes the results and stresses the need for further research. The previous review from Ricci-Cabello et al. [11] covered publications up to 2007 and included 25 papers for final assessment; just two of these are included in our review as well [34,36]. This little overlap is also due to the fact that the two reviews differ in a number of ways: The review from Ricci-Cabello et al. [11] also includes studies that define social inequalities by gender and ethnic group, that study inequalities in diagnosis of DM, and it includes four studies that are restricted to type 1 DM.

Adding some recommendations for further research, we would like to highlight eight topics: First, despite the great variety of proposals regarding appropriate assessment methods [16,41,42], quality of diabetes care should be measured in a way that enables benchmarking and comparison. A standard set of indicators for assessing good quality health care should be provided. Process indicators may not be a good predictor of patient outcome; thus, it would be better to assess patient outcomes directly [43]. For example, concerning intermediate outcome indicators, the same cut-off value should be used in all studies, preferably based on current international guidelines. This is easier said than done, of course; there are still different guidelines. Concerning cut-off points for ‘poor glycaemic control’, for example, it has been proposed to use HbA1c values above 6.5% [14] or 7% [13]. The National Diabetes Quality Improvement Alliance [44] suggests HbA1c levels above 9%. In addition, there are different opinions regarding cut-off points, especially for the elderly [12]. The standard set should also include outcome indicators.

Second, it is important to distinguish clearly between the individual and the regional level. Assessing SES at the individual level highlights the following problem: health care for people with Type 2 DM could be influenced by a number of individual risks and resources such as poor health behaviour or good social support. It would be important to know which of these risks (or resources) are especially strong (or weak) among patients with low SES and how their risks could be reduced and their resources improved. Assessing SES by regional deprivation points to another problem: resources for people with Type 2 DM could differ between regions. There could be differences concerning health care (e.g. availability of specialized physicians) and other factors such as facilities for physical activities. Important questions would be, for example: What kind of resources are especially weak in more deprived regions, and how could they be improved? What role could be played by improving health care? Also, it is possible that the proportion of patients with low individual SES is especially high in the most deprived regions, that the differences between regions could largely be explained by these individual level differences. Therefore it is necessary to differentiate clearly between effects at the individual and at the regional level, i.e. to conduct multilevel analyses. These analyses are well known in public health research [45], but we are not aware of a study on social and regional inequalities in health care for patients with Type 2 DM that is based on this kind of analysis.

Third, some associations with low individual SES seem to be fairly well established, concerning process indicators (e.g. less frequent measurement of HbA1c and lipids), intermediate outcome indicators (e.g. higher prevalence of HbA1c values above the proposed level) and diabetes-specific complications (e.g. higher prevalence of retinopathy). Some associations with regional deprivation seem to be fairly well established as well, concerning intermediate outcome indicators (e.g. HbA1c values) and diabetes-specific complications (e.g. retinopathy). The potential reasons behind these associations are poorly understood however. They may relate to social differences in training, awareness of early symptoms and compliance [40,46,47], for example, but the ‘web of causation’ has not yet been described in any detail. Health outcomes are influenced by many factors, and health behaviour plays an important role in explaining social differences [48]. The influence of health services may be limited [41], but it would be important to study the interplay between health care, on the one hand, and health behaviour, diabetes self-management and the achievement of clinical targets, on the other, especially among low SES patients.

Fourth, meta-analyses could be important especially for assessing effects that are relatively small. It is rather difficult to merge the empirical information from different studies (as mentioned above), but the chances to conduct meta-analyses should be explored in more detail. Fifth, registries provide an excellent database for these studies, but they often lack information on individual SES such as educational level or income. It is recommended that this information is added wherever possible. Sixth, different countries have different systems of health promotion and health care (e.g. concerning co-payments), and the problems and causes of social inequalities in health care may differ as well. One implication is that policy recommendations for a specific country will have to be based on empirical studies conducted in this country. Another implication is that cross-country comparisons are difficult to conduct, as all these differences will have to be taken into account, but these comparisons offer an important chance for studying the associations between system characteristics and health care inequalities. Seventh, health care for patients with type 2 DM is similar to health care for patients with type 1 DM, but the two groups of DM patients are quite different. It would be important to add a review focused on patients with type 1 DM. Last but not least, there is a great need for intervention studies, i.e. studies that compare different interventions aimed at reducing social differences in health care for people with Type 2 DM.

## Conclusion

The available evidence clearly points to the fact that socio-economic inequalities in diabetes care do exist. Low individual SES and regional deprivation are often associated with worse process indicators of care and worse intermediate outcomes (e.g. poor glycaemic, lipid and BP control), resulting in higher risks of microvascular (e.g. retinopathy) and macrovascular complications (e.g. myocardial infarction, stroke). These differences exist even in countries with a health care system that is clearly based on solidarity between all socio-economic groups (e.g. Germany). Much is known about good quality diabetes care; it is important to study in more detail why patients with low SES profit less from this knowledge.

## Abbreviations

ADA: American diabetic association; BMI: Body mass index; BP: Blood pressure; CVD: Cardiovascular disease; EPHPP: Effective public health practice project; GIS: Geographic information system; GP: General practitioner; HbA1c: Glycolised haemoglobin; IDF: International Diabetes Federation; IMD: Index of multiple deprivation; IQR: Interquartile range; LDL: Low-density lipoprotein; OECD: Organisation for economic cooperation and development; SES: Socio-economic status; Type 1 DM: Type 1 diabetes mellitus; Type 2 DM: Type 2 diabetes mellitus; UKPDS: United Kingdom prospective diabetes study; WHO: World Health Organization; ZQ: Zip quality.

## Competing interests

The authors declare that they have no competing interest.

## Authors’ contributions

All authors jointly defined the research question and the search strategy. OG and WM carried out the literature search, information extraction and first assessment. In case of disagreement a joint decision was reached together with AM. OG drafted the manuscript. All authors read and approved the final manuscript.

## Acknowledgements

This work was partially supported by the Kompetenznetz Diabetes mellitus (Competence Network for Diabetes mellitus) funded by the Federal Ministry of Education and Research (FKZ 01GI1104A).
